# Research on fault diagnosis system for belt conveyor based on internet of things and the LightGBM model

**DOI:** 10.1371/journal.pone.0277352

**Published:** 2023-03-13

**Authors:** Meng Wang, Kejun Shen, Caiwang Tai, Qiaofeng Zhang, Zongwei Yang, Chengbin Guo

**Affiliations:** 1 College of Mining, Liaoning Technical University, Fuxin, Liaoning, China; 2 School of Civil Engineering, Wuhan University, Wuhan, Hubei, China; 3 Mixlinker Networks (Shenzhen) Inc, Shenzhen, Guangzhou, China; Asia University, TAIWAN

## Abstract

As an equipment failure that often occurs in coal production and transportation, belt conveyor failure usually requires many human and material resources to be identified and diagnosed. Therefore, it is urgent to improve the efficiency of fault identification, and this paper combines the internet of things (IoT) platform and the Light Gradient Boosting Machine (LGBM) model to establish a fault diagnosis system for the belt conveyor. Firstly, selecting and installing sensors for the belt conveyor to collect the running data. Secondly, connecting the sensor and the Aprus adapter and configuring the script language on the client side of the IoT platform. This step enables the collected data to be uploaded to the client side of the IoT platform, where the data can be counted and visualized. Finally, the LGBM model is built to diagnose the conveyor faults, and the evaluation index and K-fold cross-validation prove the model’s effectiveness. In addition, after the system was established and debugged, it was applied in practical mine engineering for three months. The field test results show: (1) The client of the IoT can well receive the data uploaded by the sensor and present the data in the form of a graph. (2) The LGBM model has a high accuracy. In the test, the model accurately detected faults, including belt deviation, belt slipping, and belt tearing, which happened twice, two times, one time and one time, respectively, as well as timely gaving warnings to the client and effectively avoiding subsequent accidents. This application shows that the fault diagnosis system of belt conveyors can accurately diagnose and identify belt conveyor failure in the coal production process and improve the intelligent management of coal mines.

## 1. Introduction

As an essential transportation tool for coal production [1], the belt conveyor has the advantages of long transportation distance, large transportation volume, and sustainable transportation, it is widely used in many aspects of coal production. However, after a long time of high-load and high-intensity operations, the belt conveyor can easily occur failures such as conveyor belt deviation and slippage. These failures will not only have a significant impact on coal production but also lead to safety accidents [2]. For example, on November 1st, 2000, the second belt conveyor of Pinghu coal mine, Fengcheng Mining Bureau, Jiangxi Province, occurred due to belt friction, which caused 14 deaths and 18 injuries and more than 2 million yuan of economic loss [3]. Therefore, it is essential to ensure the safe operation of the belt conveyor. The traditional method of maintaining the safety of belt conveyors is arranging for staff to conduct periodic inspections while the belt conveyors are operating. This method can reduce the probability of accidents to a certain extent. However, the efficiency is not ideal. Therefore, new detection methods must be used to solve this problem. Developing IoT technology and intelligent algorithms provide new ideas and ways to solve this problem. Many industries have successfully used IoT technology and intelligent algorithms to solve engineering problems, and it provides samples for fault identification and diagnosis of belt conveyors in coal production.

During the Chinese 13th Five-Year Plan period (2016–2020), native coal production is required to develop in the direction of intelligence [4]. Many scholars have studied the intelligence of the belt conveyor. Based on the support vector machine algorithm, Xiangong Li et al. established an algorithmic model with 19 fault values, such as motor power, motor temperature, and CST temperature as model parameters to identify the fault of the belt conveyor [5]. The results showed that the accuracy of conveyor fault identification reached 92.7%. Mengchao Zhang et al. designed and proposed an improved Yolov3 algorithm and used it to detect the type of conveyor belt damage [6]. The experiment suggests that the improved algorithm has higher accuracy, and faster speed than the original algorithm. S. Ravikumar et al. [7] studied the faults of the belt conveyor centre and combined shaft. Taking the original vibration signal of the conveyor as the characteristic value of the K-star algorithm, the fault classification of the conveyor by the K-star algorithm was realized, and the correct classification rate for conveyer defects was 91.7%. Xiangwei Liu et al. [8] introduces an original framework of integrated maintenance decision-making for belt conveyor idlers. It realized the interaction between the theoretical estimation of reliability and condition monitoring data. Zhongyi Li et al. [9] used computer vision and statistics-related technology to monitor the operation of the belt conveyor. Wei Chen et al. [10] discusses the principle and advantages of the wireless sensor network and design an intelligent monitoring system for coal mine via wireless sensor. Shuvashis Dey et al. [11] presented a monitoring methodology that is based on UHF chipped and chipless PFID sensors, it provides a real-time monitoring schemes for conveyor belt health paraments. As can be seen from the above example, the intelligent system of belt converors is imperfect. References [5–8] establish a model through machine learning to identify belt conveyor faults. References [9–11] built a conveyor monitoring system using sensors. However, these experimental results cannot provide real-time feedback on the operational status of the belt conveyor. Therefore, the Internet of Things platform and the LGBM algorithm model were adopted in this paper to establish a fault diagnosis system for the belt conveyor. By using signal acquisition, signal transmission, and other technical means to realize the real-time collection of the belt conveyor operating status parameters and the output of diagnosis results, the monitoring and online diagnosis of belt conveyor running status was proposed in this paper.

The main contributions of this paper are as follows:

We use IoT technology to build a system to monitor and diagnose conveyor’s faults to realize complex environmental data collection. The Aprus adapter replaces the central controller to connect with the sensor. The advantages of the Aprus adapter are that it supports the data integration of various external sensors and can match the interfaces and communication protocols of most sensors on the market. At the same time, the data visualization function of the IoT facilitates the use of managers without programming experience. We provide the design process for the construction of the monitoring system.

The rest of this paper is organized as follows: Section 2 establishes and verifies the model. Section 3 introduces the structure construction process of the IoT monitoring system. Section 4 display a field test. Section 5 concludes the paper.

## 2. Research on the model of belt conveyor

### 2.1 Introduction to the LGBM model

Gradient Boosting Decision Tree (GBDT) is a commonly used model in machine learning. LGBM is a framework for implementing the GBDT model. To solve the fault diagnosis problem of the belt conveyor, the paper adopted LGBM, an effective data classification method used in a range of areas, including industry, medicine and economy [12, 13]. LGBM model based on the GBDT learning model combines the Histogram algorithm, the Gradient-based One-Side Sampling (GOSS) algorithm, and the Exclusive Feature Bundling (EFB) algorithm. It can improve the learning efficiency of the model while guaranteeing the model accuracy [14, 15].

The LGBM algorithm is as follows:

Firstly, assuming that there is a training set X = {*x*1, *x*2…*x*n}, the LGBM model will initialize a tree as a constant:

$$y_{i}^{\left(0\right)}=f_{0}=0$$ (1)

where $y_{i}^{\left(t\right)}$ is the prediction of the *i*-th example at iteration *t*.

Then the next tree is trained through the minimum loss function:

$$f_{t}(x_{i})=\text{arg}\;\underset{f_{{}_{t}}}{\text{min}}L_{\left(t\right)}=\text{arg}\;\text{min}\;L(y_{i},y_{i}^{\left(t-1\right)}+f_{t}(x_{i}))$$ (2)

where *f*_*i*_(*x*_*i*_) represents the learning model of the *t*-th decision tree

Then the next model is predicted as:

$${\text{y}}_{i}^{t}=y_{i}^{\left(t-1\right)}+f(x_{i})$$ (3)

Eqs (2) and (3) are repeated until the model reaches the termination condition. The final model formula is:

$$y_{i}=\sum_{t=0}^{M-1}f_{t}(x_{i})$$ (4)

where *M* is the number of iterations.

### 2.2 Determination of model parameters

Belt conveyors have common faults in deviation, belt breaking, slipping, and fire [16]. Deviation of the conveyor belt is generally caused by uneven force on both sides of the conveyor belt or the roller is not parallel to the centre line of the conveyor belt [17]. The conveyor belt slipping refers to the abnormal relative movement caused by the mismatching of running speed between the driving drum and the belt; this is generally due to the insufficient tension of the belt or the small friction coefficient between the roller surface and the conveyor belt [18]. When the conveyor deviates and slips, on the one hand, the conveyor belt will deviate from the original running track, so the speed of the conveyor belt will change [19]. The relative sliding of the conveyor belt will cause friction and heating, leading to the heating of the roller and the conveyor belt; if this situation is not detected in time, the belt will catch fire once the temperature rises to the ignition temperature of the conveyor belt [20]. Conveyor belt tearing and breaking means that the conveyor cannot perform a regular operation when a part of the conveyor belt is destroyed; it may be caused by friction with sensitive materials during the conveyor operation [21]. When the conveyor belt is broken, the internal tension at the break will show a specific proportional relationship with the deformation of the steel core rope. When the conveyor is running normally, the deformation of the steel rope core regularly increases with the increase of the internal tension of the belt. Once the conveyor belt joint is damaged or the tension exceeds the limit value, the steel rope core will undergo plastic deformation and no longer have the mechanical characteristics before plastic deformation; this induces changes in tension and deformation [22]. Therefore, during the conveyor operation, the conveyor belt’s joint tension can be monitored to warn against the belt breaking. When the conveyor does not work normally, the current of the conveyor motor will be disturbed. According to the failure characteristics of the conveyor, this paper selects the conveyor belt speed, motor temperature, motor current, drum temperature, and belt tension as the characteristic parameters of the LGBM model.

### 2.3 The source and preprocessing of sample data

**Fig 1:**
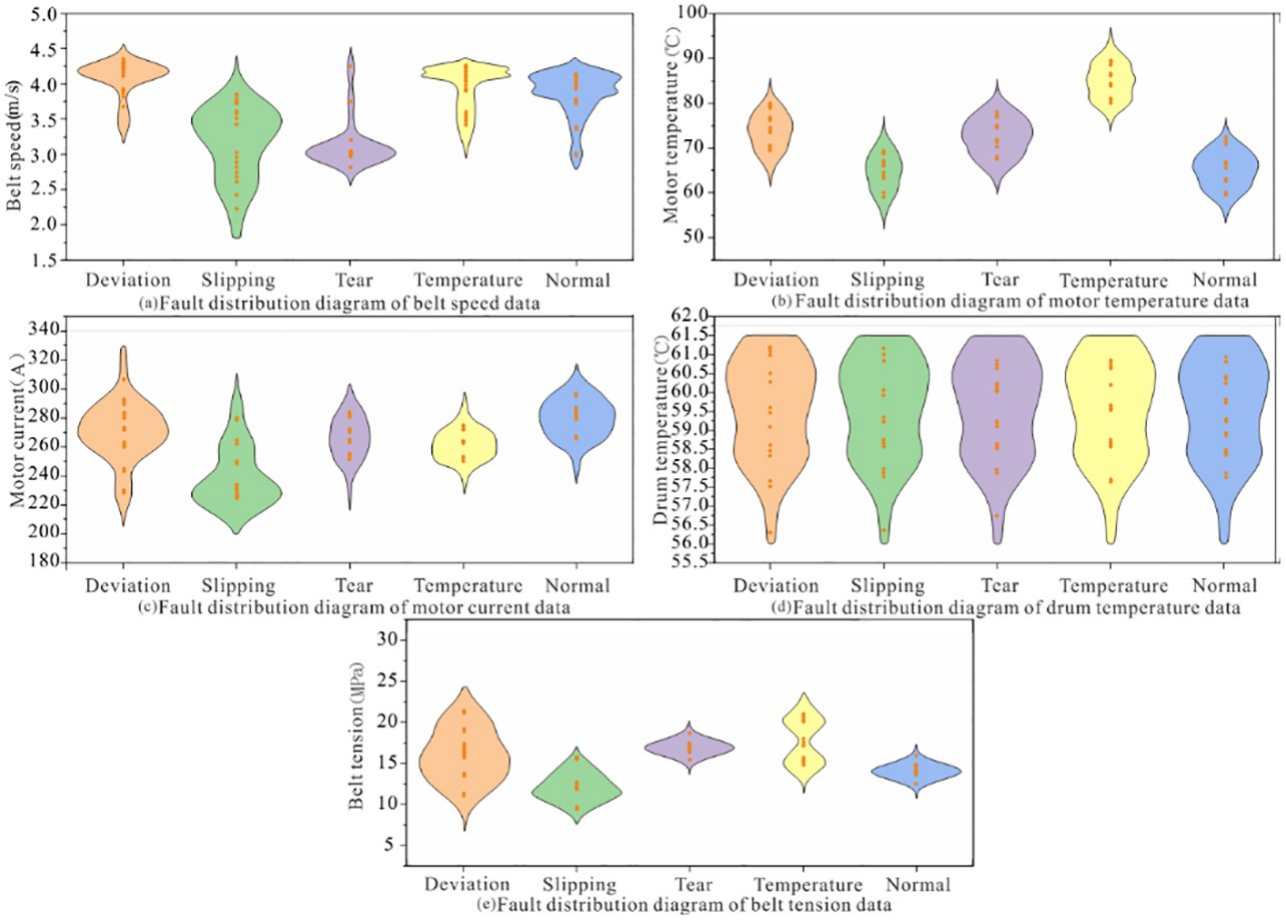
Data distribution. (a) Fault distribution diagram of belt speed data, (b) Fault distribution diagram of motor temperature data, (c) Fault distribution diagram of motor current data, (d) Fault distribution diagram of drum temperature data, and (e) Fault distribution diagram of belt tension data.

In order to train the model, this paper uses the historical belt conveyor monitoring data from a coal mine as the sample. A total of 150 sets of representative belt conveyor operating data were obtained, with 30 sets of data for each operating state. The data distribution is shown in Fig 1, and the violin’s width represents the data distribution’s density. It can be seen from Fig 1 that the distribution of each feature value on each label is relatively uniform without outliers. In order to achieve model training, 120 groups of data are randomly selected as the training set from sample data, and the rest data are used as the test set to verify the model’s accuracy. Each data set includes five features: belt speed, motor temperature, motor current, drum temperature, and belt tension. Due to the extensive data, only part of the data is displayed here. The original data is stored on the data sharing website [23]. Part of the data is shown in Table 1. Since the LGBM model supports feature classification, it is unnecessary to transform and normalize the data in the preprocessing stage [24]. Therefore, this paper does not normalize the sample data before training and testing.

**Table 1:** Partial data display.

Sample	Belt Speed m/s	Motor Tem /°C	Motor Current /A	Belt Tension /MPa	Drum Tem /°C	State
1	4.30	52.4	255.4	22	61.4	Belt broke
2	4.23	52.2	266.2	24	58.5	Belt broke
3	3.22	51.6	210.4	14	59.6	deviation
4	3.21	51.9	210.8	14	58.2	deviation
5	4.23	91.5	210.6	11	61.4	High Tem
……	……	……	……	……	……	……
150	4.25	52.3	209.7	14	59.6	Normal

### 2.4 The selection of the model performance evaluation index

The diagnosis of belt conveyor fault is a multi-classification problem. In the model training and predicting process, the performance evaluation index of the model can intuitively reflect the reasonability of the classifier, so the appropriate performance evaluation index is the key to obtaining the optimal classifier [25]. These evaluation indicators, including accuracy, error, recall, and ROC curve, are typical in binary classification problems [26]. For multi-class problems, these evaluation indicators are typical of Macro-accuracy, Macro-error rate, Macro-precision, Macro-recall, macro F-measure and macro Matthews correlation coefficient [27].

Macro-Accuracy and Macro-Error Rate have paired indicators, which respectively refer to the proportion of correctly predicted and incorrectly predicted samples, whose calculations are shown in formulas (5) to (6). The value of Macro-Accuracy and Macro-Error Rate are both in the range of [0, 1]. The closer the Macro-Accuracy is to 1, the better the model’s performance. On the contrary, the closer the Macro-Error Rate is to 0, the better the model’s performance.

$$Macro-Accuracy=\frac{1}{n}•\sum_{i=1}^{n}\frac{TP_{i}+TN_{i}}{N_{isample}}$$ (5)

$$Macro-ErrorRate=\frac{1}{n}\sum_{i=1}^{n}\frac{FP_{i}+FN_{i}}{N_{isample}}$$ (6)

where *FP*_*i*_ is in the *i*-th sample data, the number of samples predicted to be false positive; *FN*_*i*_ is in the *i*-th sample data, the number of samples predicted to be false negatives; *TP*_*i*_ is in the *i*-th sample data, the number of samples predicted to be true positive; *TN*_*i*_ is in the *i*-th sample data, the number of samples predicted to be true negative; *n* is Number of categories; *N*_*isample*_ is the total number of samples of class I sample data.

Macro-Precision and Macro-Recall, respectively, refer to the proportion of correctly predicted positive samples in the prediction results of the predicted positive samples and the actual positive samples. The calculation formula is expressed in Eqs (7) to (8). The value of Macro-Precision and Macro-Recall is in the range of [0, 1], and the closer their values are to 1, the better performance of the model.


$$Macro-\text{Pr}ecision=\frac{1}{n}•\sum_{i=1}^{n}\frac{TP_{{}_{i}}}{TP_{i}+FP_{i}}$$ (7)

$$Macro-\text{Re}call=\frac{1}{n}•\sum_{i=1}^{n}\frac{TP_{i}}{TP_{i}+TN_{i}}$$ (8)

Macro-Accuracy, Macro-Error Rate, Macro-Precision, and Macro-Recall are common and accurate evaluation indicators in multi-class models. At the same time, the performance evaluation indicators of the above model can be used to evaluate a real-time prediction model [26]. Thus, this paper chooses them as the evaluation indicators of the LGBM model.

### 2.5 Establishing and optimizing the model

In machine learning, to train and test the model, it is generally necessary to divide the sample data into a training set and a test set, where the ratio of the training set to the test set is generally 7:3 or 8:2 [28]. Based on it, according to the amount of sample data obtained and the characteristics of the model, the sample data is randomly divided into the training set and test set according to 8:2.

In the model training process, in order to obtain the optimal model, it is generally necessary to adjust and optimize the model parameters. These model parameter optimization methods, such as the grid search method, random search method, and Bayesian optimization algorithm, are common parameter optimization methods in machine learning. Among them, grid search is essentially an enumeration method, which determines the optimal value by finding all the points within the search range. Although this search method consumes lots of computing resources, the method is mature and stable, and the model result obtained by this method generally has high accuracy. Thus, this method has been widely used in the parameter optimization of intelligent models. The random search means randomly selecting sample points in the search range. Its theoretical basis is that if the sample point set is large enough, the optimal global value can be found with high probability. The Bayesian optimization algorithm finds the parameters that improve the objective function to the optimal global value by learning the shape of the objective function.

To find out the optimal hyperparameters, this paper use grid search method, random search method, and Bayesian optimization algorithm to optimize the model parameters. By comparing these results to select proper method, the results of model parameter optimization by three methods are shown in Table 2. It can be seen that Grid search and Bayesian optimization algorithm have similar precision, but grid search spend less time, Random search has uncertainty, and the experimental results differ greatly. Considering comprehensively, this paper chooses the grid search method to optimize the super parameters. After optimization of model parameters, the training time is 4.75s and the prediction time is 0.68s.

**Table 2:** Model parameters.

Parameter	Gird	Random	Bayesian	Boundary	Step
Max_depth	6	20	25	(-1,10)	1
Num_leaves	50	42	48	(1,100)	10
Learning_rate	0.05	0.00475	0.026	(0.001,1)	0.005
N_estimators	1500	800	178	(100,2000)	100
Memory space	24.793MB	38.906MB	7.586MB		
Running time	10.351s	6.850s	55.737s		

Max_depth represents the depth of the decision tree model. The greater the value, the greater the accuracy, but the higher the risk of overfitting. Num_leaves represents the maximum number of leaves on a tree, and its increase can improve the training set’s accuracy and the chance of injury from overfitting. According to the documentation, a simple method is num_leaves = 2^(max_depth). Objective represents the model type, parameter Multiclass represents the target is a multi-classification task, Learning_rate represents the learning rate, which determines the convergence rate of the model, and N_estimators represents the number of model iterations [29].

### 2.6 Evaluation of the model

K-fold cross-validation is a commonly used method to evaluate the performance of a model. This method divides the sample data into K parts in equal proportions and selects one part of the data as the test sample data and the remaining K-1 parts as the training sample data. This process is a test. Then, the selected sample data is put back to re-select new data as new test sample data and the remaining K-1 data as training sample data; this process is repeated K times, it can effectively avoid model overfitting due to the small scale of sample data set, and the K value is generally selected as 3, 5, and 10. Since the sample data in this paper is not large, K = 5 is selected, that is, five-fold cross-validation. This paper uses five-fold cross-validation to test the model [28]. The confusion matrix is often used to calculate various performance indicators in classification problems and reflects the model’s overall performance. Therefore, the confusion matrix is used to calculate the model performance indicators of this model. After verification and calculation, the prediction accuracy rates of each fold test are 97%, 95%, 98%, 97%, and 96%, respectively. The final output of the confusion matrix is shown in Table 3.

**Table 3:** Confusion matrix output result table.

Actual vs. Predicted	1	2	3	4	5
1	18	0	0	0	0
2	0	14	0	0	0
3	0	0	13	0	0
4	0	0	1	15	0
5	0	0	0	0	12

In Table 3, each column represents the predicted category, each row represents the actual category of the data, and the value on the diagonal represents the number of correctly predicted samples. It can be seen from Table 3 that the values on the off-diagonal lines are close to 0, indicating that the number of correctly predicted samples for each category is relatively close to 100%, and the predicted results are in line with the actual results. It can be seen from Table 4 that the precision rate, recall rate, and F1-score coefficient of each feature value are close to 1, indicating that the classifier of this model has good performance. According to the model’s test results and the model evaluation indicators, it can be known that the LGBM model can achieve high accuracy after sufficient training and is an ideal model.

**Table 4:** Evaluation index values.

	Precision	recall	F1-score	support
Slipping	1.00	1.00	1.00	18
Belt broken	1.00	1.00	1.00	14
Normal	0.93	1.00	0.96	15
deviation	0.88	0.88	0.93	16
High temperature	1.00	1.00	1.00	12
Accuracy			0.96	75
Macro-avg	0.98	0.97	0.96	75
Weighted avg	0.98	0.98	0.96	75

## 3. Establishing of the fault monitoring and diagnosis system of the belt conveyor

### 3.1 Introduction to the Internet of Things

The Internet of Things refers to establishing a network that enables smart devices in an organisational information system to connect another and exchange data with central storage, and the communication model is mainly a publication-subscription mechanism [30]. In 1999, Professor Kevin Ashton first proposed the concept of IoT [31]. In recent years, the IoT has been applied to all walks of life as a new technology.

### 3.2 Choice of Internet of Things platform

Shenzhen smart IoT Network Co., Ltd., founded in 2014, is one of China’s earliest industrial Internet solution providers. MixIoT is an IoT underlying system independently developed by Shenzhen smart IoT Network Co., Ltd. This company provides a primary underlying platform for various IoT scenarios and solutions, which makes it possible to conduct secondary development of the IoT platform according to actual needs. At the same time, MixIoT is also a flexible and open IoT system, and any device can be quickly connected to it to realize the intelligence of complex factories through configuration. At the same time, it provides data collection, data processing, data storage, and data application interfaces; besides, it also supports business applications such as message push, fault alarm, data report, operation and maintenance, and work order processing. More importantly, it supports a standard API interface and provides third-party development [32]. In addition, MixIoT has a complete security protection system, with security mechanisms in data collection, transmission and application stages to prevent data from flowing to malware [33].

Currently, the mainstream IoT in the market include Baidu IoT Hub, Alibaba Cloud IoT, QQ IoT, and other platforms. The functions provided by the MixIoT platform for third-party developers are not available on other IoT platforms. Therefore, it is needed to design the required functions according to an actual requirement,, this article chooses the MixIoT platform as the building platform for the system.

### 3.3 Selection of hardware devices

**Fig 2:**
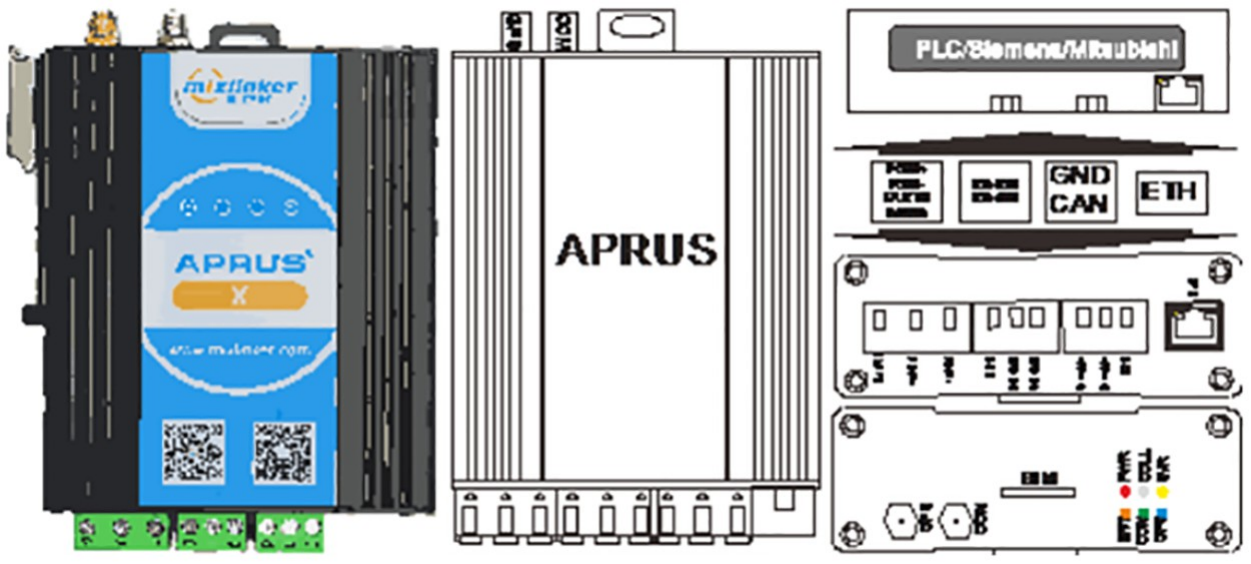
Aprus adapter.

Aprus (Advanced Programmable Remote Utility Server) adapter is an IoT adapter for industrial equipment developed by Zhiwulian Company. It supports a video camera, data integration of external temperature, humidity, and infrared sensors, and supports these interface types such as RS232, RS485, CAN, and Siemens PLC. The interface and appearance of the adapter are shown in Fig 2. It can efficiently provide IoT solutions for industrial equipment and form a professional industrial IoT adapter for the designed application mode. Using the Aprus adapter is straightforward and easy to connect with the device, allowing customers to redefine the data acquisition logic and data acquisition protocol of Aprus through the LUA script. It can also realize web configuration LUA script, download, remote upgrade, and redefine. At the same time, the adapter can also report and log sensor faults or send commands to the device to modify parameters. These functions enable the Aprus adapter to realize complex industrial equipment collection scenarios. In the fault diagnosis system for the belt conveyor, the function of the Aprus adapter is equivalent to a central controller. It uses the RS-485 interface to connect the sensor and then reads the data collected by the sensor. One Aprus adapter can be connected to multiple sensors. In addition, the Aprus adapter is also equipped with a particular communication card, which ensures the network communication between the Aprus adapter and the human-machine interface of the IoT platform.

The fault diagnosis in the system is based on the data collected by sensors, and collecting these data requires laying appropriate sensors. According to the model training test in Section 2.2, the sample data features are belt speed, motor temperature, motor current, drum temperature, and belt tension. Therefore, the sensors selected in this paper include encoders, temperature sensors, tension sensors, and motor drives. The peripheral linear speed of the driven drum can be approximately considered to be equal to the speed of the conveyor belt, and the encoder can be installed on both sides of the driven drum to monitor the rotational speed of the driven drum. The temperature sensor is installed on the motor and the roller side to monitor the motor and the roller’s temperature. The tension sensor monitors the tension of the conveyor belt joint, and the motor driver monitors the motor. The sensor parameters are shown in Table 5. There are differences in interfaces and communication protocols between different types of sensors. Syntax and semantic conflicts between data sources will impact data integration [34]. This paper selects sensors with the same interface and communication protocol to avoid this impact.

**Table 5:** The parameters of sensor technical.

	The encoder	Temperature sensor	Tension sensor	Motor drive
Precision	±0.1%	±0.1°C	±1MPa	0.1A
Baud rate	9600bps			
Interface	RS485			
Communication Protocol	Modbus			

### 3.4 System design and construction

#### 3.4.1 System framework

**Fig 3:**
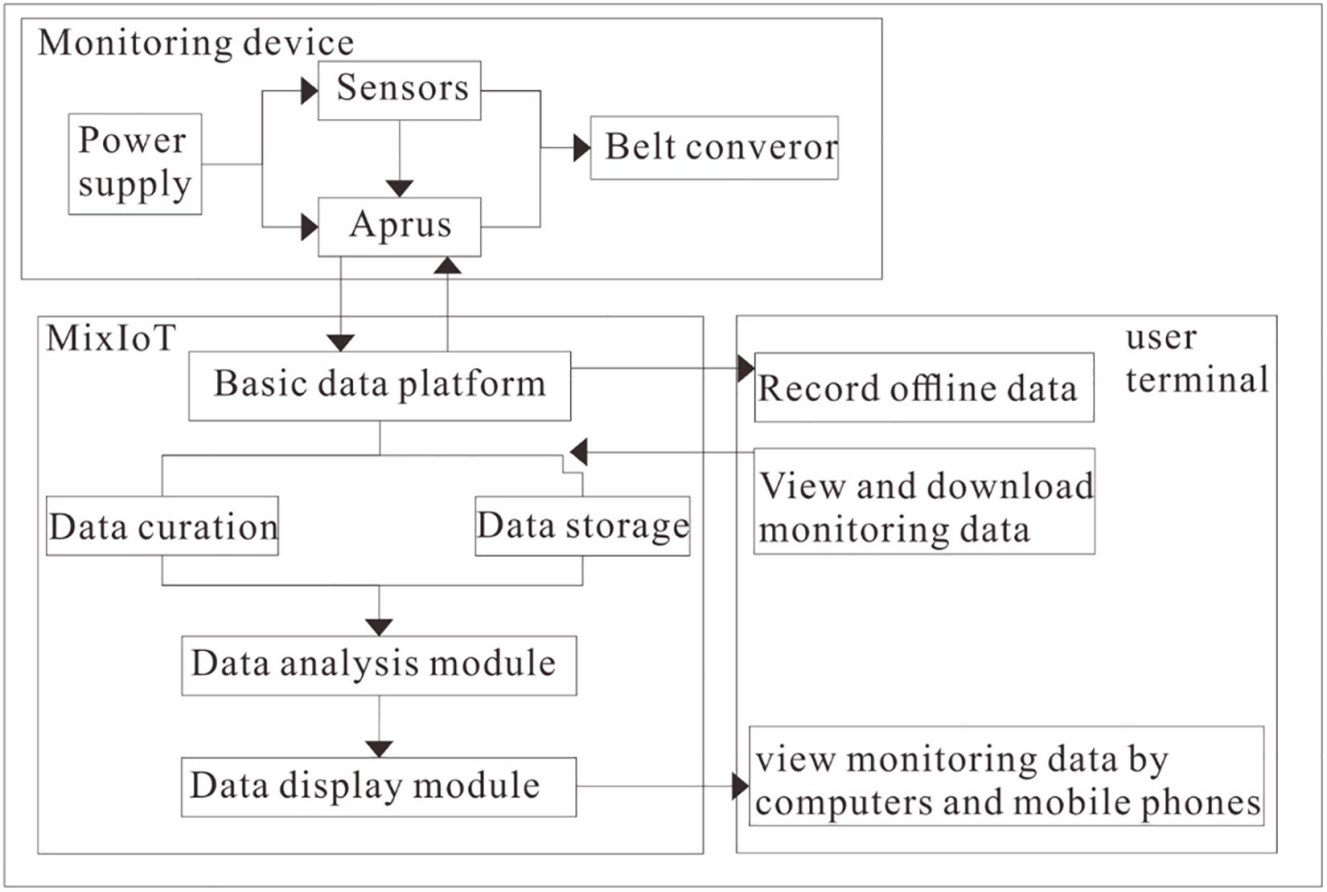
The framework for system design and composition.

The framework for system design and composition is shown in Fig 3.

The components and functions of the belt conveyor fault diagnosis system are as follows:

1. Monitoring device: the leading equipment is a conveyor, temperature sensor, tension sensor, encoder, motor drive and Aprus adapter. The power supply, adapters and sensors are installed at the conveyor’s corresponding positions to complete the belt conveyor’s monitor. A variety of sensors is a data source in the monitoring system, which connects and exchanges data with the Aprus adapters. Then, these exchanged data are loaded to the back-end data platform of the Internet of Things in the form of 2G/4G/WIFI.
2. Data platform: these exchanged data are transmitted to the MixIOT platform through the network. Then the platform collects various monitoring data, correlates and stores real-time monitoring data, and ensures that the monitoring system has good data management capabilities; At the same time, the monitoring data, equipment status and other information are displayed visually on the page.
3. User side: users can remotely monitor data in real-time, manage historical data, and understand the self-status, sensor status and location of multiple monitoring nodes. At the same time, users should be responsible for data upload and storage, statistical management, subsequent sensor increase and decrease management, and monitoring node setup and installation.

#### 3.4.2 Connection between device and platform

Chapter 3.2 introduces that the Aprus adapter and the IoT platform maintain network communication through the communication card, and the data transmission between them relies on the communication protocol and the LUA scripting language. Based on the network communication between the Aprus adapter and the Internet of Things platform, the LUA scripting language can be written in the Internet of Things platform. The company provides a template for the LUA scripting language, which only needs to reset the corresponding number for the data collected by the sensor and input some parameters of the sensor, the acquisition node part, and the reporting node part. The parameters to be transferred in the interface attribute of the parameter object include: baud rate, data bit, stop bit, and check bit of the sensor interface. The objects of the acquisition node include Modbus ID, address, function code, data length, and interval time (ms). For each sensor node, the objects of the reporting node include Modbus ID, function code, slave address, reporting data type, register address, reporting cycle, and reporting data label. Sensors with different communication protocols and interfaces only need to configure the corresponding parameters to connect to the MixIOT platform.

### 3.5 The embedment of the model

Python is a simple, efficient, object-oriented programming language, and its interpreted nature makes it widely used for scripting and rapidly developing an application on most platforms. The MixIoT is developed in C++. Boost::python and python C API can embed models written in Python into MixIoT. The essence of model embedding is to embed Python into C++. Boost::python dramatically simplifies the task of embedding Python, but it can not fully embed python modules into C++ wrapper libraries, so much of the work must be done through the Python C API. Firstly, the boost library needs to be built in the main program to initialize the python interpreter. Then, the Python C API adds the search path of the python module to the Python interpreter, and once the search path is added, the PyImport_Imp-ortModule function can load the Python module. After initializing the python interpreter, the main module is imported, and the namespace is resolved, which results in a blank runtime environment where we can call Python code and add modules and variables.

### 3.6 Working procedure of the belt conveyor

**Fig 4:**
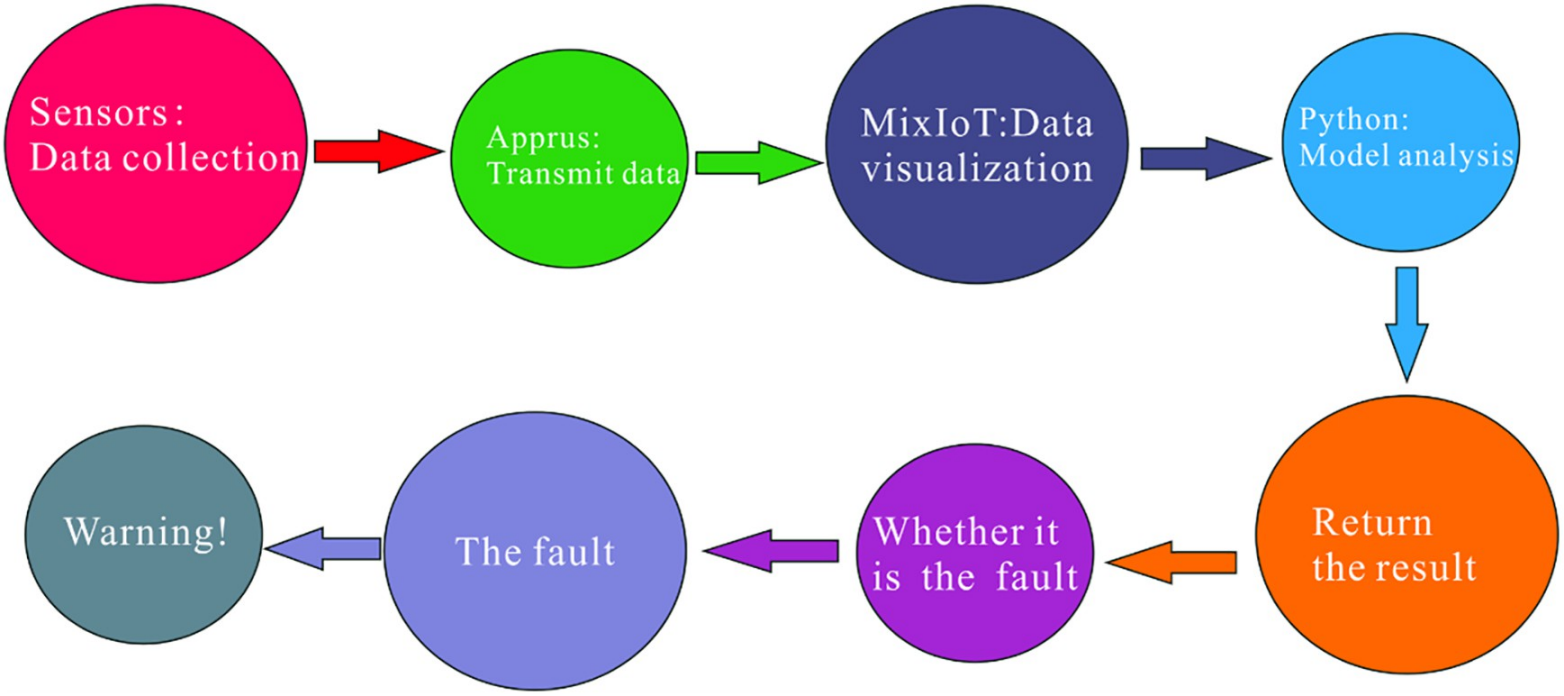
Work flow chart.

The fault diagnosis system for the belt conveyor is mainly composed of a belt conveyor, sensor, Aprus adapter, and MixIoT platform. The main function of the sensor is to collect the operating status parameters of the belt conveyor and transmit this data to the Aprus adapter. The Aprus adapter is the bridge between the sensor and the MixIoT platform. Firstly, the Aprus adapter collects and organizes sensor data within a specific range and then transmits the collected data to the MixIoT platform. Secondly, real-time monitoring and statistical calculation of data can be performed on the MixIoT platform. Finally, analyzing and judging the data by the model and then returning the diagnostic value of the model to the MixIoT platform. If the platform receives information about the model failure, an alarm window will pop up, and the staff can remotely shut down the conveyor to prevent secondary accidents. The system workflow is shown in Fig 4.

## 4. Display and application of the platform

### 4.1 Display of the platform

**Fig 5:**
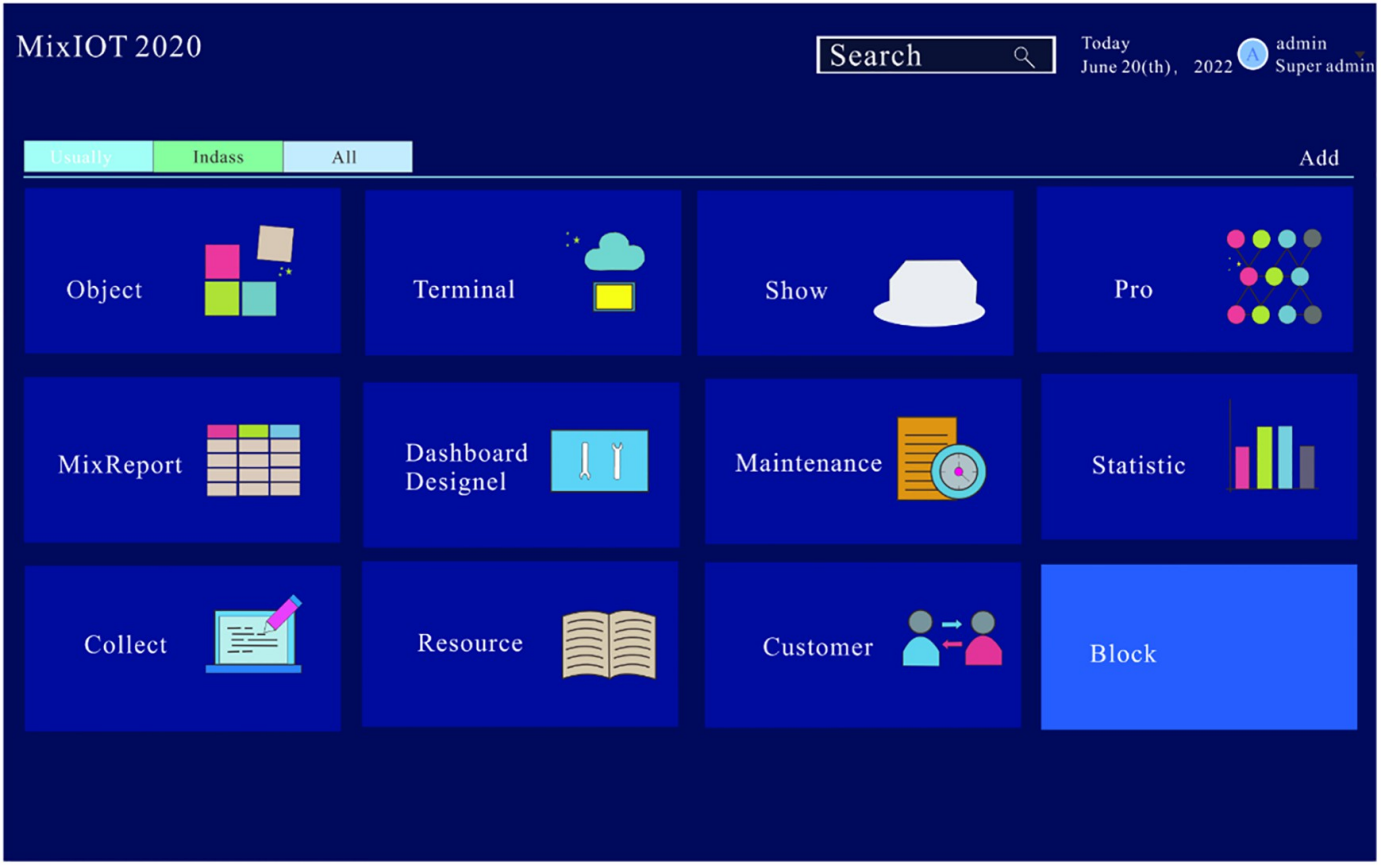
MixIoT platform.

The URL of the MixIoT platform is introduced into the browser to enter the login interface of the platform and enter the account and password to access the platform. Fidis is Mixiot’s SaaS application platform, which supports MixIoT interface operation management. The platform home page is the portal navigation that shows a series of tabs; Each tab is an independent application, and clicking on a tab will jump to the tab application. The platform page is shown in Fig 5.

We can know the functions of each tab from Fig 5, in which the object management provides the data visualization function; in the object management tab, we can know the dynamic change graph of the data collected by the sensor. The management tab provides design functions, and the operator can design the data visualization interface according to their preferences; the collect tab is the storage of historical data.

### 4.2 Application of the system

#### 4.2.1 The profile of the mine

Although the basic framework of the fault diagnosis system for the belt conveyor has been constructed and can theoretically meet the Operation requirements, the actual operation effect still needs to be verified on site, So this paper applies the system to a coal mine in Shanxi Province, China, which is an open-pit coal mine; the minefield is generally an irregular polygon with a length is about 2.6 km and a width of about 0.8 km. The thickness of the coal seam is 0.8~2.4 m, the average thickness is 1.60 m, and the buried depth is about 163 m. The semi-continuous mining process is adopted: single bucket-truck-semi-fixed crushing station-belt conveyor-dumper. Among them, the width of the working face belt conveyor is B = 1600 mm, the belt speed V = 4.2m/s, the length L = 1286m, the horizontal section is 864m, and the slope section is 422m. Some horizontal sections are selected as the application site of the fault diagnosis system for the belt conveyor.

#### 4.2.2 Application effect and analysis

**Fig 6:**
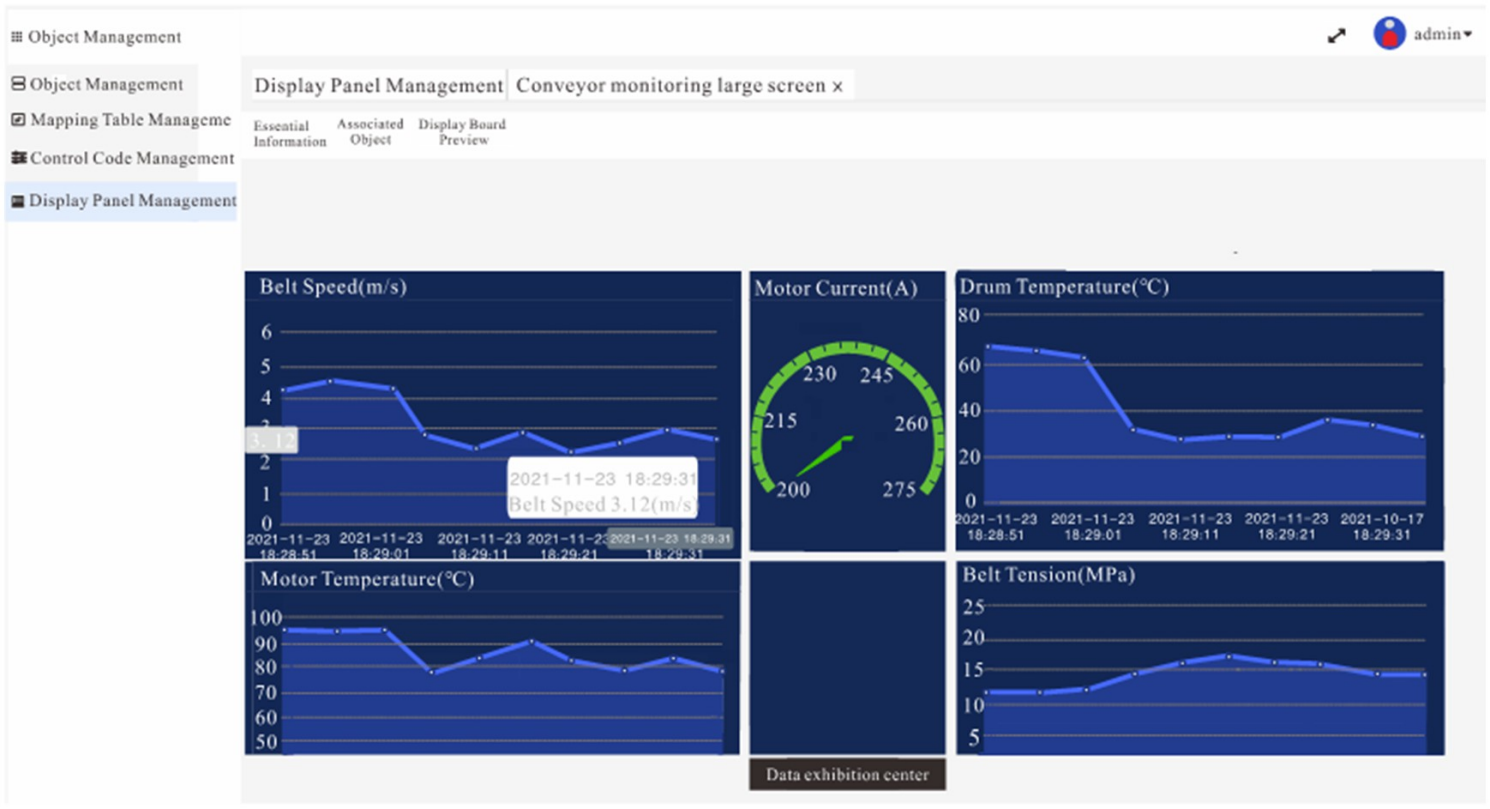
Remote monitoring screen.

**Fig 7:**
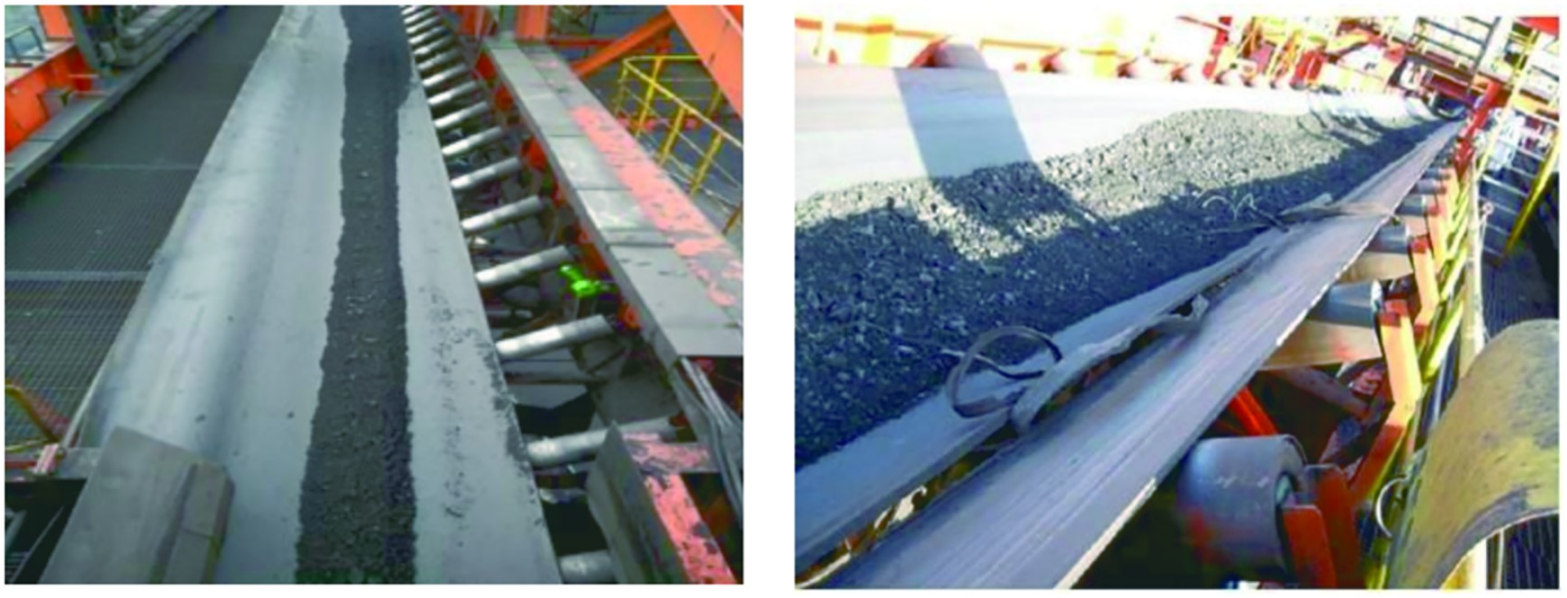
Conveyor failure diagram.

**Fig 8:**
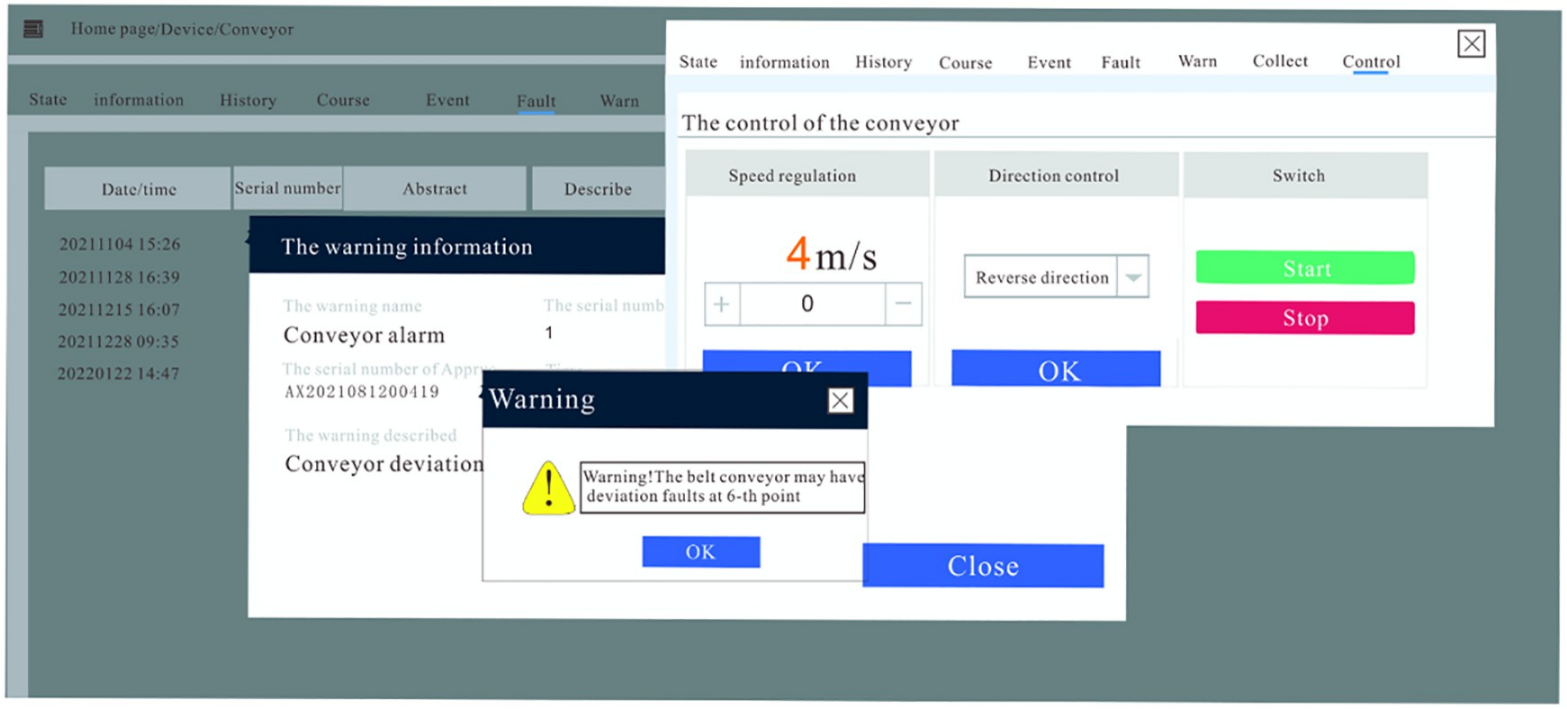
Platform warning diagram.

After the fault diagnosis system for the belt conveyor was applied in a mine, the data collection of various operating parameters of the belt conveyor was conducted, and the LGBM model was used to diagnose the faults of the conveyor online. Fig 6 shows a data visualization of the belt conveyor operating parameters. It can be seen that the operating parameters are changing drastically, indicating that some types of failure have occurred in the belt conveyor; In the first three months since the fault diagnosis system was applied, there were four faults, including two deviations, one slip, and one tear. During this process, four faults were accurately identified and diagnosed. Fig 7 is a diagram of the failure of the belt conveyor, the left picture is the deviation diagram of the belt conveyor, and the right picture is the tearing diagram of the belt conveyor. Fig 8 is an early warning diagram of the MixIoT platform. We can see the alarm information of the belt conveyor from the interface and can also realize the remote control of the conveyor by inputting parameters.

### 4.3 Data analysis

**Fig 9:**
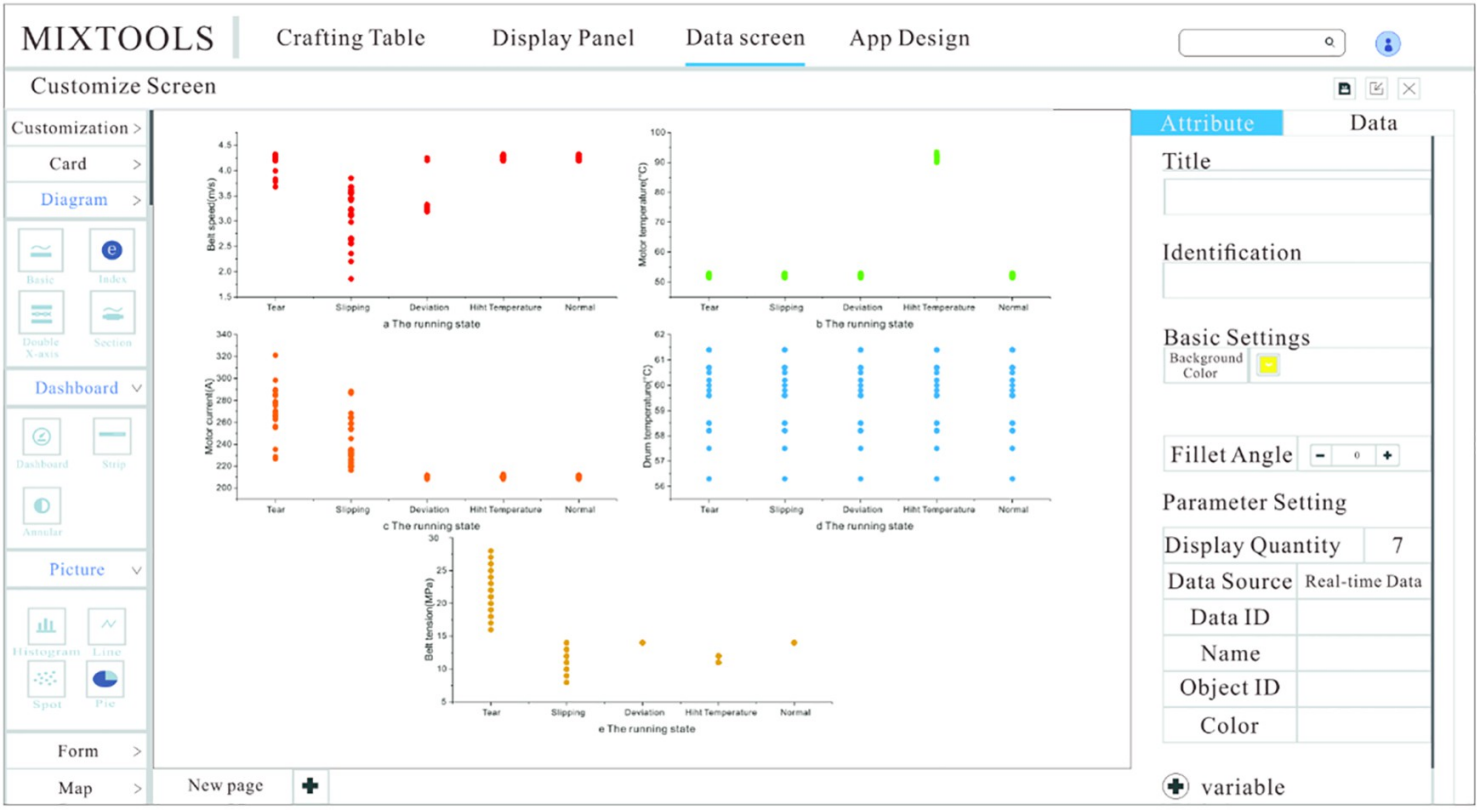
Distribution of characteristic data.

The data tab can analyze the stored data. Fig 9 shows the data distribution of tag values concerning characteristic values. It can be seen from Fig 9 that the data distribution of each tag value on the characteristic value is different, indicating that different types of faults have different effects on the belt characteristics. Based on the analysis of belt speed data, it can be seen from Fig 9 that when the belt speed is 4.0~4.5m/s, the conveyor belt is in a normal state. When the conveyor deviates, the belt speed data is distributed between 4.0~4.5m/s and 3.0~3.5m/s, which shows that the deviation fault occurs rapidly. On the contrary, the occurrence of slip fault is gentle, and the belt speed is 1.75–3.75m/s. The high temperature does not affect the belt speed in a short time.

## 5. Conclusion and prospect

### 5.1 Conclusion

1. This paper used the sensor and MixIoT platform to build a conveyor remote monitoring system; thus, the remote monitoring of the conveyor is realized. At the same time, the Aprus adapter communicates with the host computer through the communication card and simplifies the connection between the traditional industry and the host computer through the bus.
2. Using the operating parameters of the belt conveyor to establish the LGBM model, which can accurately diagnose the fault of the belt conveyor.
3. After putting the fault diagnosis system for the belt conveyor into practical application, it is found that the MixIoT platform can observe the value change of each parameter during the operation of the belt conveyor in time. At the same time, when the conveyor fails, it can receive the feedback of the model in time and issue a warning. These results show that the fault diagnosis system for the belt conveyor not only realizes the remote monitoring of the belt conveyor but also realizes the online diagnosis of the belt conveyor, indicating that the MixIoT platform can provide timely feedback on the diagnosis results of the model.
4. The existing conveyor fault diagnosis system generally combines LabView and matable, and is a self-programming framework. Compared with the existing projects, the Internet-based belt conveyor fault diagnosis system has the following advantages: (i) Big data framework: to process massive data, the conveyor monitoring system should have a robust data framework. (ii) Data analysis: through the data analysis function, we can better understand the situation of the conveyor and analyze the characteristics of conveyor failures. MixIoT itself can analyze data without relying on other software. (iii) Security: MixIoT has protection mechanisms in data acquisition, transmission layer, application layer and data exchange layer to prevent data from being sent to unauthorized places.

### 5.2 Prospect

At present, the domestic coal industry is building a smart mining system. As a part of the intelligent mine system, the belt conveyor fault diagnosis system should first consider compatibility with other subsystems. For example, the conveyor fault diagnosis system and the intelligent rapid tunnelling system coexist as a system, working independently and mutually. Secondly, we need to improve the conveyor fault diagnosis system. More features can provide a better understanding of the problem and a better analysis of the problem. We will consider adding sound and vibration features in the following research step. However, the sound and vibration eigenvalues cannot be expressed in digital form. The next challenge is how to use algorithms to integrate the two types of eigenvalues of digital and acoustic signals and express them in the Internet of Things.
